# Impact of endobronchial allergen provocation on macrophage phenotype in asthmatics

**DOI:** 10.1186/1471-2172-15-12

**Published:** 2014-03-10

**Authors:** Carla Winkler, Lena Witte, Natali Moraw, Conny Faulenbach, Meike Müller, Olaf Holz, Frank Schaumann, Jens M Hohlfeld

**Affiliations:** 1 Department of Respiratory Medicine, Hannover Medical School, Hannover, Germany; 2 Fraunhofer Institute for Toxicology and Experimental Medicine, Hannover, Germany; 3 Biomedical Research in Endstage and Obstructive Lung Disease Hannover (BREATH), Member of the German Center for Lung Research, Hannover, Germany

**Keywords:** Asthma, M2 macrophages, Endobronchial allergen provocation, Segmental allergen challenge

## Abstract

**Background:**

The role of M2 polarized macrophages (MΦ) during the allergic airway inflammation has been discussed in various animal models. However, their presence and relevance during the chronic and acute phase of allergic airway inflammation in humans has not been fully elucidated so far. In the present study we phenotypically characterized macrophages with regard to M2 polarization in mice, a human *in vitro* and a human *ex vivo* model with primary lung cells after endobronchial provocation.

**Results:**

Macrophages remained polarized beyond clearance of the acute allergic airway inflammation in mice. Alveolar macrophages of asthmatics revealed increased mRNA expression of CCL13, CCL17 and CLEC10A in response to allergen challenge as well as increased surface expression of CD86. Further, mRNA expression of CCL13, CCL17, and CLEC10A was increased in asthmatics at baseline compared to healthy subjects. The mRNA expression of CCL17 and CLEC10A correlated significantly with the degree of eosinophilia (each P < .01). Furthermore, macrophages from asthmatics released significant amounts of CCL17 protein *in vitro* which was also found increased in BAL fluid after allergen provocation.

**Conclusions:**

This study supports previous findings of M2 macrophage polarization in asthmatic subjects during the acute course of the allergic inflammation and provides evidence for their contribution to the Th2 inflammation.

## Background

Pulmonary dendritic cells, activated Th2 effector cells, and their respective cytokine and chemokine networks in the sensitization and initiation phase of allergic airway inflammation have been explored intensively [1-3]. The question, whether the most abundant resident cell type in the alveolar space, the macrophage, plays a significant role in these processes has been neglected in this setting for years [4]. Increasing knowledge about macrophage polarization brought alveolar macrophages back into focus. In the context of allergic airway inflammation the contribution of the alternatively activated M2 phenotype with its specific functionality appears to be of special interest [5].

M2 polarization encompasses at least two subtypes M2 and M2-like macrophages dependent on the cytokine milieu macrophages are subjected to. M2 macrophages differentiate in response to IL-4 and IL-13 whereas M2-like macrophages acquire their phenotype in response to TGF-b, IL-10 or PGE and additional TLR activation [6]. M2-like macrophages release high amounts of IL-10 and are thus considered to be anti-inflammatory. However, M2 macrophages are functionally considered to promote clearance of parasite infections [7], they are involved in tumor progression [8], and they contribute to tissue remodeling [9]. Markers for M2 macrophages differ between mice and man, with Ym1, Fizz1, Arg specifically described to be up-regulated in mice [10], whereas expression of the mannose receptor CD206 was defined for M2 macrophages from both species [11,12]. In humans, several markers for M2 polarization have been described such as up-regulation of HLA-DR and increased expression of Th2 chemokines CCL17, CCL18, CCL22 and CCL24 [13]. Importantly, most data on macrophage polarization are derived from animal studies. M2 markers between mice and man are not identical, and our understanding of the characteristics of M2 polarization in human asthma is still incomplete [14]. Recently, Staples and colleagues published an important paper in which they phenotypically characterized M2 polarization of alveolar macrophages in a group of non-asthmatic subjects and asthmatic patients [15]. Using the currently accepted panel of human M2 markers, they showed a partial M2 polarization of sputum and BAL macrophages in symptom-free asthmatics. Interestingly, the CCR4 ligand CCL17, a key chemokine for the recruitment of CCR4^+^ effector cells, was prominently induced, indicating that M2 macrophages might also facilitate the inflammatory immune response in humans.

With the current study we extended the phenotypic and functional characterization of alveolar macrophages in asthmatic patients, by paying special attention to the late phase of allergic airway inflammation. Therefore, we investigated macrophage polarization before and after endobronchial allergen challenge. As significant levels of IL-4 and IL-13 are predominantly present in the acute effector phase of the allergen response, we first assessed the kinetics of macrophage polarization in a murine asthma model beyond the acute phase of inflammation. To study M2 polarization, we further established a human *in vitro* model with monocyte-derived macrophages (MDM) of atopic donors. Subsequently, human alveolar macrophages isolated from non-asthmatic and mild asthmatic subjects undergoing an endobronchial allergen challenge were analyzed. Two doses of allergen were used in separate lobes of the subjects in order to investigate the impact of the severity of the inflammatory response on the extent of polarization. Our findings confirm and extend previous findings and provide data about the plasticity of macrophages in mild asthmatic subjects under stable non-inflamed conditions and during the late phase of the allergic airway inflammation.

## Results

### M2 polarization persists after resolution of the acute allergic airway inflammation in mice

**Figure 1:**
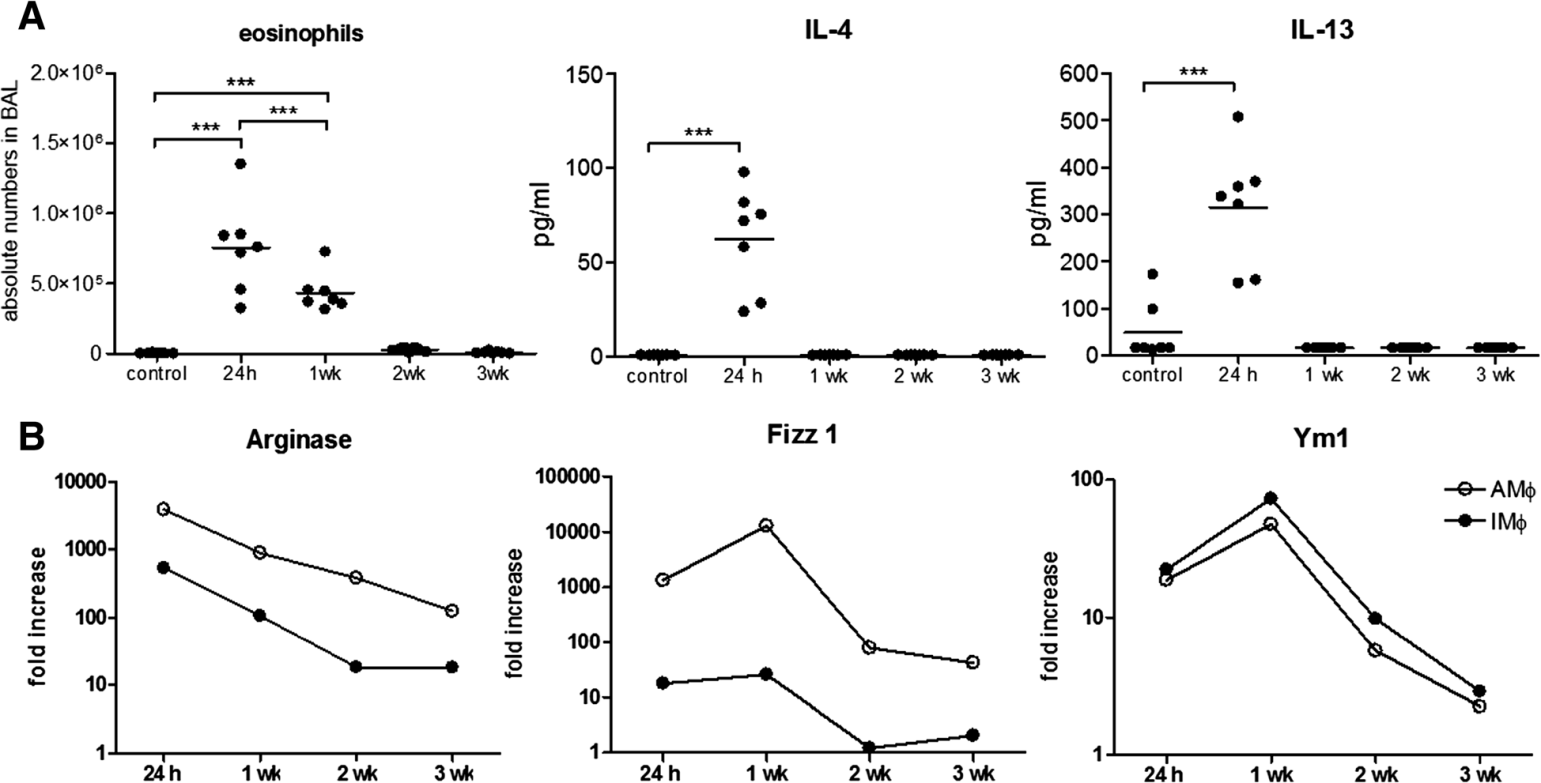
**Kinetics of M2 polarization in mice after the induction of an acute allergic airway inflammation.** Alveolar (AMΦ, open circles) and interstitial (IMΦ, closed circles) macrophages were isolated 1 day (24 h), 1 week (wk), 2 wk and 3 wk after ovalbumin aerosol provocation. **A**, Eosinophil numbers, IL-4 and IL-13 levels (n = 7). Data were analyzed with one way ANOVA. *** P < .001. **B**, Gene expression of M2 markers Arginase, Fizz1, and YM1 are presented as fold induction compared to PBS treated mice (each data point represents n = 5 pooled animals).

To evaluate the time course of alternative activation of pulmonary macrophages a mouse model of acute allergic airway inflammation was used to study the expression of M2 marker genes in alveolar and interstitial lung macrophages. The mRNA expression of previously described murine M2 markers (Arg, Fizz1 and Ym1) and iNOS (M1 marker) was analyzed over a period of three weeks after the induction of acute inflammation in mice. The inflammation kinetic assessed by inflammatory cell influx and cytokine levels in BAL revealed elevated eosinophils 24 h and 1 week after allergen provocation (P < .001), whereas IL-4 and IL-13 levels in BAL were only increased during the peak of the acute inflammation at 24 h (P < .001) (Figure 1, A). Messenger RNA expression of Arg, Fizz1, and Ym1 was markedly increased in alveolar and interstitial macrophages of ovalbumin sensitized animals compared to the control group (Figure 1, B) whereas iNOS mRNA expression was not altered (data not shown). The expression of M2 marker genes declined over time, but after three weeks AMΦ still revealed a higher expression of these genes compared to the control group. Interestingly, the level of expression for the 3 genes in IMΦ and AMΦ constantly fell to 3.3 ± 0.3% at 3 weeks compared to 24 h.

### In vitro M2 polarization of human derived macrophages

**Figure 2:**
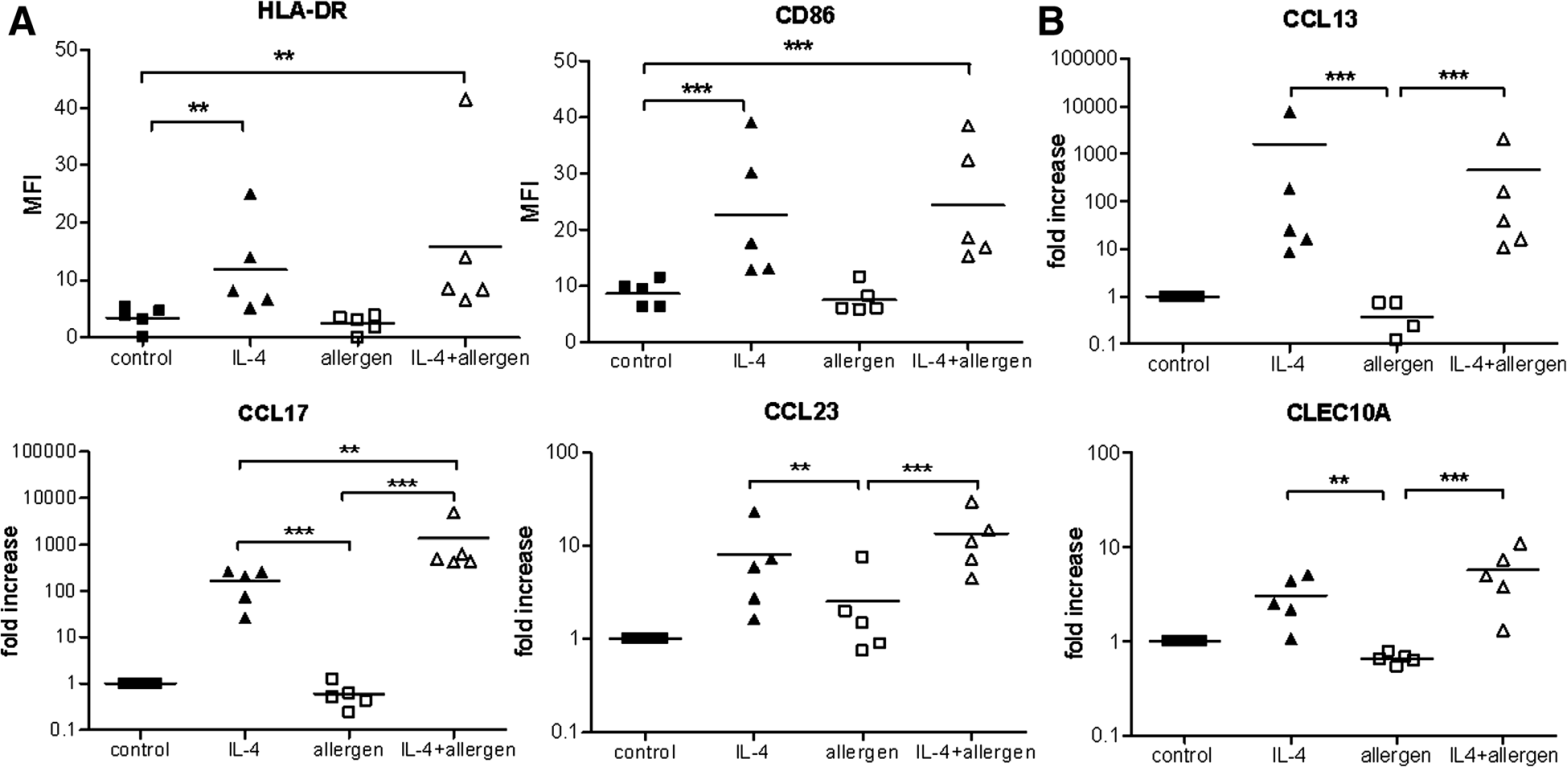
**Characterization of human monocyte-derived macrophages which were alternatively activated in the presence of IL-4 and allergen. A**, Surface marker expression of HLA-DR and CD86 measured by flow cytometry after 48 h of polarization as mean fluorescence intensity (MFI). **B**, mRNA expression of M2 markers measured by RT-PCR as fold induction compared with control macrophages. Bars indicate mean values; data were analyzed with one way ANOVA (n = 5). **P < .01, and *** P < .001.

To characterize M2 markers in human cells in more detail, macrophages were generated from peripheral blood monocytes of atopic subjects and stimulated with IL-4 ± allergen. Co-stimulatory surface markers like HLA-DR and CD86 were significantly up-regulated on the surface of macrophages in the presence of IL-4 (P < .01) compared to control macrophages (Figure 2, A). Protein expression of the mannose receptor (CD206) was not regulated by IL-4 (data not shown). Compared to control macrophages, the mRNA expression of M2 markers CCL13, CCL17, CCL18 (data not shown), CCL23 as well as CLEC10A were found to be significantly increased in the presence of IL-4 (all P < .01) (Figure 2, B). Interestingly, the mRNA expression of CCL17 was further increased when allergen was present during stimulation with IL-4 (P < .01), whereas allergen exposure during culture had no effect on the mRNA expression of other M2 markers.

### Induction of autologous lymphocyte proliferation by human MDM

As mouse M2 macrophages revealed an increase of co-stimulatory molecules and an enhanced endocytosis of soluble antigens [11], we investigated the potential of these macrophages to present antigen to autologous T-cells. Macrophages were generated from human peripheral monocytes isolated from asthmatic patients and stimulated with IL-4 and in addition with grass allergen extract prior to co-culture with autologous lymphocytes.

**Figure 3:**
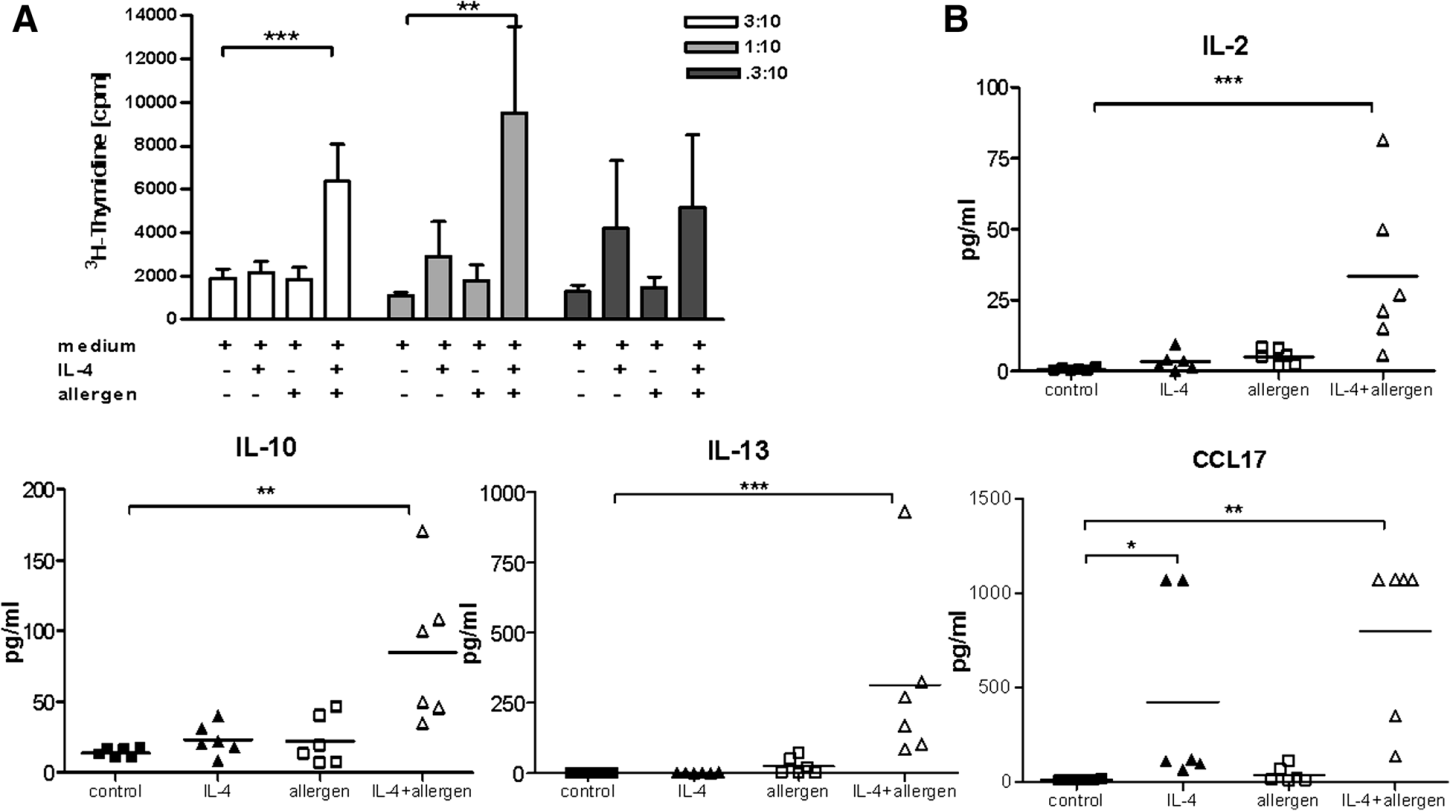
**Characterization of autologous lymphocyte proliferation by *in vitro *IL-4 polarized human monocyte-derived macrophages (MDM). A**, Induction of proliferation by IL-4 polarized macrophages ± grass allergen extract. Three macrophage/lymphocytes ratios are shown (mean ± SEM, n = 8). **B**, Levels of cytokines/chemokines in supernatants of co-cultures (n = 6). Bars indicate mean values. Data were analyzed with one way ANOVA. *P < .05, ** P < .01, and *** P < .001.

Native macrophages, not stimulated with IL-4, did not induce any T-cell proliferation in contrast to M2 (IL-4 stimulated) macrophages which induced low but significant induction of proliferation when primed with allergen (P < .01) (Figure 3, A). In comparison to professional antigen presenting dendritic cells (generated in the same model), M2 macrophages revealed a five-fold lower capacity to induce specific T-cell proliferation (data not shown).

Analysis of cytokines in the supernatant after IL-4 stimulation revealed increased amounts of CCL17 compared to control MΦ. Furthermore, macrophages induced Th2 cytokine release like IL-2, IL-10 and IL-13 by lymphocytes when stimulated with IL-4 and allergen (Figure 3, B). In contrast, TNF-α, CCL24, and CCL22 release were found unchanged compared to medium control macrophages (data not shown).

### M2 polarization of human AMΦ after endobronchial allergen challenge

Next, we investigated the extent of macrophage polarization after endobronchial allergen challenge in healthy and asthmatic subjects (Table 1). The inflammatory response in the control and allergen challenged segments was assessed by cell differentiation and cytokine measurements of the recovered BAL fluid. The two segments that received the standard allergen dose were similar with respect to the influx of eosinophils (Table 2). A lower percentage of eosinophils was found in the segment that received 1/10 of the standard allergen dose (P < .01). Compared to baseline, levels of IL-5 in BAL increased after allergen provocation (P < .001), however with no significant differences between the allergen doses (Additional file 1: Figure S1). For further analysis, cells and supernatants from the two standard allergen segments were pooled. On protein level, CCL17 and CCL22 were found to be increased due to endobronchial allergen challenge with differences between allergen doses (Additional file 1: Figure S1) (P < .01).

**Table 1:** Patient characteristics

	**Healthy**	**Asthmatic**
No.	4	11
Sex (male/female)	4/0	9/2
Age (y)	45(40–50)	43(37-50)
Atopy	no	yes
FEV1 (% predicted)	108.3(103.8-11.2)	94(83.1-110.7)
FEV1/FVC ratio	79.6(75.1-80.3)	76.2(70.8-86.7)
Metacholine PC_20_	n.d.	1.5(0.9-5.7)
Allergen (BU/ml)	100	62.5(62.5-125)
IgE (U/ml)	23(9.5-68)	173(97–262)

Median and interquartile ranges (IQR) are given.

**Table 2:** Bronchoalveolar lavage data

**Segment**	**Recovery (ml)**	**Total cells (x 10**^**6**^**)**	**Macrophages (%)**	**Eosinophils (%)**	**Neutrophils (%)**	**Lymphocytes (%)**	**Epithelial cells (%)**
**B***(H)*	49 (35-58.8)	2.1 (1.5-3.6)	91 (88.3-92.9)	0	1 (0.6-1.8)	7.4 (4.5-9.8)	0.8 (0.5-1.8)
**S***(H)*	73.5 (66.5-78)	7.7 (4.4-15.3)	88.9 (61.2-92)	0.3 (0-0.6)	2.4 (1.2-31)	5 (3.4-8.3)	0.7 (0.2-2.3)
**A**_**1/10**_*(H)*	68.5 (64-78)	5.4 (5.3-6.2)	86.7 (76.1-93.4)	0.5 (0-1.6)	6.5 (1.6-12.5)	6.6 (2.5-10.3)	0.1(0-2.8)
**A1***(H)*	55.5 (47-61)	3.5 (2.7-5.5)	79.4 (73.7-86.3)	0.2 (0.4)	7.8 (3.3-11.3)	7.8 (3.3-10.4)	0.6 (0.3-1.1)
**B***(A)*	52.0 (44-60)	4.7 (1.2-5.7)	91.3 (81-93.8)	0.3 (.0.5)	2 (0.3-2.3)	5.8 (3-12)	1 (0.8-2.5)
**S***(A)*	55 (51-69)	5.6 (2.6-9.5)	88.3 (84.8-91)	0.3 (0-1.3)	3.8 (1.3-9.8)	5 (3.3-7.8)	0.8 (0.3-2)
**A**_**1/10**_*(A)*	54 (47-63)	6 (3.9-14.2)	70.3 (29-84.5)	7.8 (3-30.3)***§	3.5 (1.3-18.8)	3.8 (2-6.5)	1 (0.3-1.3)
**A1***(A)*	56 (48-63)	9.3 (3.9-20.9)	47.3 (31.3-63.8)^***§#^	30.8 (12.8-46)^***§#^	6.3 (1.8-9.3)	3.5 (2.5-4.8)	0 (0-0.7)
**A2***(A)*	43 (32-63)	8.1 (5-13.1)	38 (27.7-56.3^)***§#^	38.3 (10.3-63.8)^***§#^	6.5 (2.3-18)**	3.8 (2.3-6)	0.3 (0-9)

*(H)* Healthy subjects, and *(A)* asthmatic patients who underwent bronchoalveolar lavage at baseline (B) and 24 hours after instillation with saline (S), standard allergen (A), in two different segments (A1 and A2), and one-tenth of the standard allergen dose (A1/10). Median and interquartile ranges (IQR) are given. ***versus baseline P < .001, **versus baseline P < .01, § versus saline P < .001, # versus A_1/10_ P < .01

**Figure 4:**
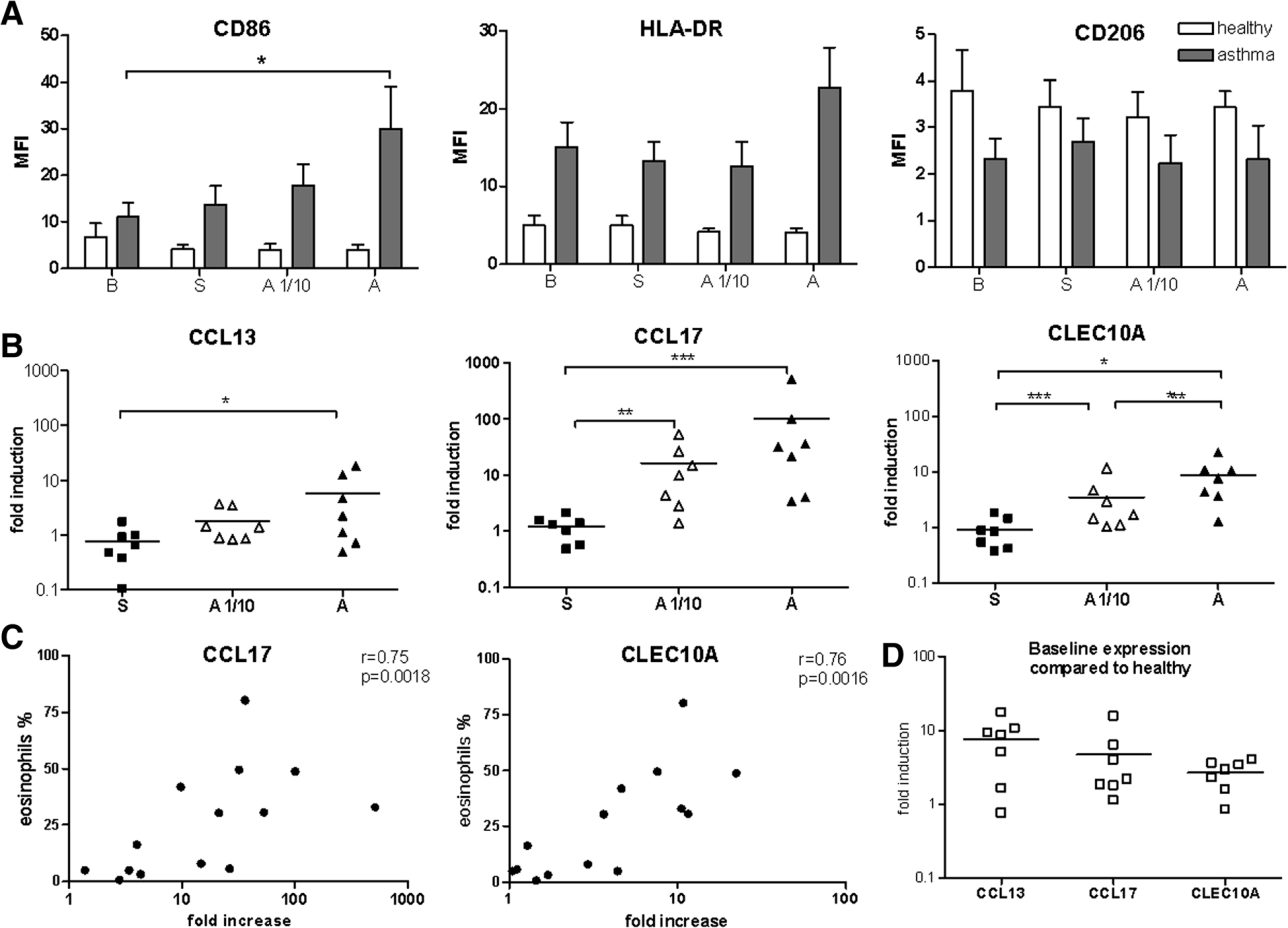
**Characterization of human alveolar macrophages (AMΦ) after endobronchial allergen provocation in healthy individuals and asthmatics.** AMΦ were isolated at baseline (B) and from saline (S), standard allergen (A) and one-tenth of the standard allergen dose (A1/10) challenged segments. **A**, Protein surface marker expression of AMΦ, mean fluorescence intensities (MFI) after isotype control correction are shown (n = 4 for healthy subjects and n = 6-8 for asthmatics). **B**, mRNA expression of CCL13, CCL17 and CLEC10A as fold induction compared to individual baseline control (n = 7). **C**, Correlation between CCL17 and CLEC10A mRNA expression and eosinophilia in BAL of allergen challenged segments (A + A1/10, n = 14). **D**, Gene expression under baseline conditions (as fold induction compared to mean baseline expression in macrophages of healthy subjects) (n = 7). Data **(A, and B)** were analyzed with one way ANOVA. *P < .05, ** P < .01 **, and *** P < .001. The Spearman rank correlation test was used in C.

The protein expression of CD86 measured by flow cytometry was significantly higher in macrophages from asthmatic patients compared to healthy subjects and further up-regulated after standard allergen provocation in asthmatic patients (P < .05) (Figure 4, A). The MFI value of surface HLA-DR was higher in asthmatic patients compared to non-asthmatic subjects but did not reach statistical significance. No differences were observed for the surface expression of CD206 (Figure 4, A).

Compared to isolated macrophages from the saline segment, the mRNA expression of CCL13, CCL17, and CLEC10A (normalized to individual baseline expression) was increased after application of the standard allergen dose, while low dose allergen challenge increased CCL17 and CLEC10A but not CCL13 (P < .05) (Figure 4, B). In contrast, the mRNA expression of CD206, CCL18, CCL23, and the M1 marker CXCL10 were not changed by allergen provocation (Additional file 2: Figure S2). In healthy subjects no alteration of M2 marker mRNA expression was observed in isolated macrophages in none of the different segments (Additional file 3: Figure S3).

The mRNA expression of CCL17 and CLEC10A (Figure 4, C) in macrophages isolated from the standard allergen and low allergen segment correlated with the percentage of eosinophils in BAL fluid (r = .76, P = .002 and r = .75, P = .002, respectively). As mRNA expression of M2 markers in healthy subjects was not altered due to allergen exposure, we normalized expression levels in macrophages from asthmatic subjects relative to mean baseline values of healthy subjects (= fold induction, Figure 4, D and Additional file 2: Figure S2). This analysis showed an increased baseline expression of CCL13, CCL17 and CLEC10A in macrophages from asthmatics patients compared to healthy subjects.

### Human AMΦ from endobronchial allergen challenged lung segments are functionally altered

According to the *in vitro* model using human MDM and autologous T-cells from peripheral blood, we evaluated the induction of antigen-specific T-cell proliferation of human AMΦ which were primed with allergen *in vivo*. Alveolar macrophages were isolated from BAL fluid by adherence and co-cultured with autologous T-cells from peripheral blood.

**Figure 5:**
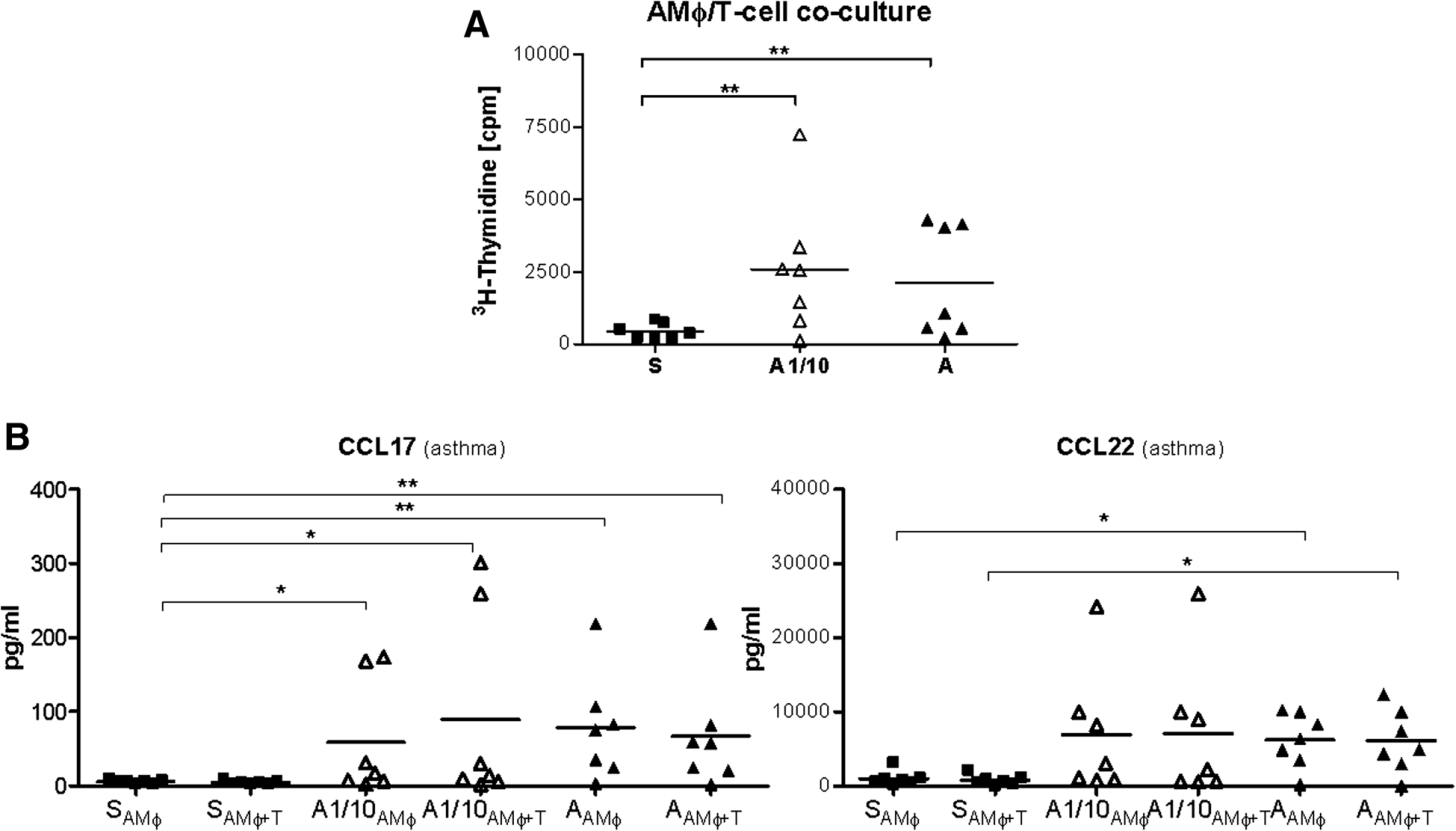
**Induction of allergen-specific T-cell proliferation by human alveolar macrophages (AMΦ) after endobronchial allergen provocation in asthmatics. A**, Proliferation of autologous CD4^+^ lymphocytes (T) after co-culture with AMΦ isolated from bronchoalveolar lavage from lung segments challenged with saline (S), one-tenth of the standard dose (A1/10) and the standard allergen dose (A), AMΦ/T-cell ratio 3:10. **B**, Chemokine levels in the supernatant of co-cultures. Bars indicate mean values (n = 7), data were analyzed by one way ANOVA. *P < .05, and **P < .01.

Mean values of T-cell proliferation were higher with macrophages isolated from the low dose and standard allergen dose challenged segments, respectively compared to the saline segment (P < .01, Figure 5, A). However, the overall magnitude of proliferation was about 10-fold lower when compared to the proliferation induced by pulmonary myeloid dendritic cells of asthmatic patients in a previous study. In this study dendritic cells were isolated from BAL after endobronchial allergen challenge by flow cytometry based cell sorting and co-cultured likewise with autologous lymphocytes [16]. Cytokine and chemokine analysis of co-culture supernatants of either AMΦ or AMΦ + T-cells showed that AMΦ isolated from asthmatics after endobronchial allergen challenge released increased amounts of CCL17 and CCL22 compared to AMΦ isolated from the saline challenged segment (P < .01, Figure 5, B). In addition increased levels of CCL17 and CLL22 were also found in BAL fluid from asthmatic patients after endobronchial allergen challenge with both allergen doses (Additional file 1: Figure S1).

In contrast to the human MDM co-culture model, no induction of Th2 cytokines by CD4^+^ T-cells was observed. Alveolar macrophages isolated from healthy subjects did not induce any proliferation nor did they release CLL17 or CLL22 (data not shown).

## Discussion

This study provides evidence for an augmented expression of CCL13, CCL17, and CLEC10A in AMΦ from patients with mild asthma during episodes of acute allergic airway inflammation. The expression of macrophage CCL17 and CLEC10A was related to the degree of eosinophilic inflammation. As increased amounts of CCL17 have been shown to attract CCR4^+^ effector cells like eosinophils or specific Th2 lymphocyte subsets [17,18], it is tempting to speculate that macrophages play an important role in modulating the degree of allergic inflammation. Our data is in line with and confirms recent results of Staples and co-workers, who showed that human AMΦ from asthmatic patients display a partial M2 polarization already under non-inflamed conditions with increased mRNA expression of CCL17, CLEC10A and protein release of CLL17 and CCL22 [15].

It has been demonstrated in mouse models that macrophages undergo M2 polarization during the acute phase of allergic airway inflammation. However, the time course of polarization markers after resolution of the acute inflammation has not been investigated. Our data demonstrate that the up-regulated expression of established murine M2 polarization markers (Ym1, Fizz1, and Arg) outlive the inflammatory response. The decline of polarization markers over time might be due to replacement by new monocytes entering the alveolar space after resolution of the cellular inflammation, which is also supported by the concordant and continuous decline of all 3 investigated M2 related genes. An impaired phagocytosis of *E. coli* by murine M2 macrophages supports the hypothesis that clearance mechanisms might be altered, making the airways more susceptible for bacterial or viral infection during this resolution period [19]. Therefore, polarized macrophages seem to be capable to play a role in sustaining an inflammatory condition after acute allergen exposure.

We found several markers of M2 polarization to be induced in response to IL-4 in human MDM such as CCL17 and CLEC10A. These polarized macrophages induced allergen-specific T-cell proliferation and Th2 cytokine secretion. Importantly, IL-4 polarized human MDM do not fully reflect the AMΦ phenotype in asthmatics, as the cytokine milieu *in vivo* is not restricted to solely M2 stimuli.

It was previously shown that IL-4 stimulation of murine macrophages *in vitro* leads to increased CCL17 release [18,20]. We could extend these findings for human AMΦ derived from allergen challenged segments as these cells express increased amounts of CCL17 on mRNA and protein level after allergen challenge. However, it remains to be elucidated to which extent macrophages account for the increased CCL17 levels in BAL of asthmatic patients since dendritic cells and epithelial cells have also been identified as prominent sources [21-24]. We did not observe CCL17 release by native T-cells of asthmatic patients in our co-cultures [25]. In opposite to findings of Staples, we found CCL17 mRNA already increased at baseline in macrophages from mild asthmatic patients compared to healthy subjects and additionally identified CCL13 to be up-regulated. CCL13 is a potent chemokine which attracts CCR3^+^ cells like eosinophils, Th2-lymphocytes, basophils and correlates with asthma exacerbation [26,27]. CCL13 protein has been shown to be increased in BAL of asthmatic patients compared to healthy controls [28]. Our mRNA results in AMΦ show that polarized macrophages represent an additional source of this protein beside epithelial cells in response to allergen provocation in the airways.

The macrophage galactose-type C-type lectin (MGL) CLEC10A which we and others found up-regulated on alveolar macrophages from asthmatic patients [15] recognizes carbohydrate structures with terminal galactose or N-acetylgalactosamine residues, which are present on naturally occurring allergens [12,29]. Additionally, the mannose receptor (CD206) [30] and IgG receptor (CD32) [31] are described to be increased on macrophages from asthmatic patients. Whether this up-regulation is capable to alter phagocytosis of M2 macrophages remains to be elucidated. Interestingly, phagocytosis of bacteria is impaired in patients with severe asthma [32,33] and IgG-opsonized yeast are less effectively cleared by macrophages from asthmatic patients [34]. Impaired phagocytosis of bacteria was also reported from various *in vitro* models of M2 polarization, also supporting the hypothesis that pulmonary clearance of inhaled pathogens might be hampered [35]. In our study, CD206 was not differentially expressed on macrophages from asthmatic patients compared to healthy subjects and not regulated in response to allergen exposure.

In asthmatic patients chitinases (chitotriosidase, CHIT1) and chitinase-like proteins (YKL-40) in serum and BAL correlate with the severity of the disease, the degree of airway remodeling, and the frequency of asthma exacerbations [36-38]. These molecules are released from activated monocytes and macrophages and are suggested to be linked with remodeling processes [39,40]. CCL18, a macrophage-derived chemokine has been described to attract T-lymphocytes and to promote collagen production by human fibroblasts [41,42]. Interestingly, we did not observe CCL18 up-regulation in alveolar macrophages 24 h after segmental allergen challenge, while we found CCL18 increased in MDM *in vitro* at 48 h after IL-4 stimulation. This is in line with a recent *in vitro* study investigating the kinetics of CCL18 induction showing maximum release at 72 h but no significant change at 24 h after stimulation with allergen [41]. In line with older reports, we found an increased expression of HLA-DR and other co-stimulatory surface markers on M2 macrophages [31,43]. Although the results from our *in vitro* allergy model show that M2 macrophages induce T-cell proliferation and the release of Th2 cytokines, we were not able to confirm the magnitude of proliferation and cytokine release in similar experiments using AMΦ. Consequently, this questions their relevance for allergen presentation. As a limitation, we did not investigate whether macrophages from asthmatic patients exhibit a suppressive function on DC-induced T-lymphocyte proliferation as has been reported before [44].

There is increasing evidence that the different models available to study the concept of macrophage polarization are providing controversial results. Murine models of allergic asthma revealed that the course of the allergic airway inflammation was not influenced by the presence or absence of IL-4 receptor-α bearing macrophages which become polarized during the allergic inflammation [45]. In contrast, the transfer of IL-4 receptor-bearing macrophages aggravated the eosinophilic inflammation in IL-4 receptor-deficient mice [46]. However, in an animal model of acute exacerbation M2 macrophages triggered Th2 cytokines in CD4^+^ T-lymphocytes through the interaction with CD80/CD86, which was not seen in a model of mild chronic asthma [47]. A rhinovirus-induced exacerbation study identified M2 macrophages after allergen challenge which subsequently released more Th2 cytokines and CCL11 after additional rhinovirus infection. On the other side, depletion of macrophages before allergen/rhinovirus challenge lead to reduced eosinophilia, CCL11 and IL-13 levels [48]. Thus, functional consequences of macrophage polarization might become more apparent during asthma exacerbation phases.

## Conclusions

The main focus of our study was the role of macrophage polarization in asthmatic patients. Taken together, we were able to show a close relationship between the degree of eosinophilic inflammation and M2 polarization, which is characterized by an increased expression of CCL13, CCL17 and CLEC10A. Together with the data from the animal and in-vitro models these data indicate that M2 macrophages might contribute to the acute inflammation by their Th2 cytokine and chemokine release. Future studies need to refine functional implications of M2 polarized macrophages in particular with pathogen-induced exacerbations and with different asthma phenotypes.

## Methods

### Kinetics of M2 marker expression in a mouse model of acute asthma

Female BALB/c mice of 6–8 weeks of age were sensitized according to the standard acute ovalbumin (ova)-model as described before [49]. Mice were killed 24 h, 1 week, 2 weeks and 3 weeks after the last ova-aerosol challenge (see methods section in the supplement). Alveolar macrophages (AMΦ) were isolated from BAL by adherence to plastic for 1 h at 37°C and 5% CO_2_. Interstitial macrophages (IMΦ) were isolated from lung digests by anti-CD11b magnetic bead separation (Miltenyi Biotech, Bergisch Gladbach, Germany) and subsequent plastic adherence to remove contaminating cells. Real time PCR of M2 marker gene expression and cytokine measurement in BAL was performed as described below.

### Clinical study design and study population

Peripheral blood derived mononuclear cells (PBMC) and autologous lymphocytes were obtained from eight atopic subjects who were allergic to grass. For isolation of AMΦ, eleven patients with mild intermittent asthma and four healthy controls underwent endobronchial allergen provocation with grass allergen extract (SQ225, Alk-Albello Arzneimittel GmbH, Hamburg, Germany) as described in detail before (see the methods section in the supplement) [50,51]. For isolation of CD4^+^ lymphocytes peripheral blood was taken one day before endobronchial allergen challenge. The clinical study was performed in compliance with the Declaration of Helsinki. The Ethics committee of Hannover Medical School approved the study (ref. #5670). All study subjects gave written consent after being fully informed about the purpose of study and the potential risks that were associated with the study procedures.

### Characterization of in vitro polarized human monocyte derived macrophages

Isolation of monocytes and lymphocytes from peripheral blood of atopic individuals was performed as described before [52]. Briefly, PBMC were isolated from whole blood by density gradient centrifugation using Ficoll-Paque PLUS (GE Healthcare Europe, Freiburg, Germany). Monocytes were isolated with anti-CD14 antibodies (Miltenyi Biotech, Bergisch Gladbach, Germany) by magnetic cell separation of PBMC and cultured for 6 days in RPMI 1640 + Glutamax (supplemented with 7% of autologous serum and 800 U/ml GM-CSF). GM-CSF was chosen for differentiation as it leads to macrophages which resemble more closely the alveolar phenotype [53]. Cells negative for CD14 were collected as lymphocytes and kept frozen until co-culture at -80°C. After differentiation to macrophages cells were alternatively activated with 10 ng/ml IL-4 for 24 h and afterwards stimulated with 2000 BU/ml grass allergen extract (SQ225, Alk-Albello Arzneimittel GmbH, Hamburg, Germany) for 48 h. Cells were harvested and splitted for either analysis of surface markers or M2 marker gene expression or further co-cultured with autologous lymphocytes.

### Isolation of human alveolar macrophages after endobronchial allergen challenge

Cells from BAL were re-suspended in culture medium with 7% autologous serum. Macrophages were isolated by plastic adherence for 1 h at 37°C and 5% CO_2_. Adherent cells were washed 3 times with PBS and either transferred to RLT-buffer (Qiagen, Hilden, Germany) for RT-PCR analysis or co-cultured with autologous lymphocytes.

### Co-culture of human macrophages and autologous CD4^+^ lymphocytes

Both, monocyte-derived macrophages and AMΦ were co-cultured with autologous lymphocytes in three or two ratios, respectively (0.3:10, 1:10 and 3:10). In detail, 0.3-3 × 10^4^ macrophages were cultured per well with 1 × 10^5^ lymphocytes in triplicates. Proliferation was assessed by ^3^H-thymidine incorporation after 5 days of culture as described before [19]. For co-culture of AMΦ autologous CD4^+^ lymphocytes were isolated by negative depletion using Dynabeads (Invitrogen, Darmstadt, Germany) from peripheral blood. An AMΦ/T-lymphocyte ratio of 1:10 and 3:10 was analyzed. For cytokine measurements, supernatants from AMΦ that were cultured for 5 days in the presence or absence of grass allergen extract (2000 BU/ml) were analyzed.

### Analysis of M2 markers by RT-PCR

RNA was extracted either from MDM or from AMΦ after plastic adherence using RNeasy Mini Kit (Qiagen, Hilden, Germany) according to the manufactures instructions. RNA transcription was performed with an Omniskript Reverse Transcription Kit (Roche, Basel, Switzerland). Real time PCR was performed with LightCycler®FastStart DNA Master^Plus^ Sybr Green 1 (Roche, Basel, Switzerland) (see the methods section in the supplement). The expression of marker genes was normalized to a house keeping gene (β-actin for mouse and GAPDH for human samples) and quantified according to the ΔΔCt-method [54].

### Analysis of cytokines from supernatants of human MDM or AMΦ, and BAL fluid

Release of cytokines to supernatants of *in vitro* cell cultures was performed by Multiplex MAP cytokines/chemokine panels (Merck Millipore, Billerica, USA). Measurement of cytokines and chemokines in BAL was performed using a Th1/Th2 10-plex from MSD (Meso Scale Discovery, Rockville, USA).

### Statistics

Statistical analyses were performed using one way Anova with Newman-Keuls post hoc testing. (GraphPad Prism Version 4; GraphPad Software, San Diego, California). Data were log-transformed if not normally distributed. Results were considered to be significant at a P value of < 0.05.

## Abbreviations

AF647: Alexa Fluor 647; AMΦ: alveolar macrophages; APC: Allophycocyanin; Arg1: Arginase 1; BAL: Bronchoalveolar lavage; CLEC10A: Macrophage galactose C-type lectin (CD301); cpm: Counts per minute; CXCL10: Interferon gamma induced protein (IP10); DC: Dendritic cells; FACS: Fluorescence-activated cell sorting; FIZZ1: Found in inflammatory zone; M1: Classically activated (macrophages); IMΦ: Interstitial macrophages; i.p.: Intraperitoneal; M2: Alternatively activated (macrophages); MDM: Monocyte-derived macrophages; ova: Ovalbumin; PE: Phycoerythrin; PBMC: Peripheral blood derived mononuclear cells; PE-Cy7: Phycoerythrin–cyanine 7; Ym1: T-lymphocyte-derived eosinophil chemotactic factor (ECF-L).

## Competing interests

The authors declare that they have no competing interests.

## Authors’ contributions

CW has made substantial contributions to conception and design, acquisition and interpretation of data and drafting the manuscript. LW carried out the sample preparation and analysis (RT-PCR, FACS and cytokines) of human pulmonary cells. NM carried out the mouse model and the MDM in vitro data. CF and FS performed the bronchoscopy study. MM contributed to the study conception and design. OH performed the statistical analysis and contributed to draft the manuscript. JMH has made substantial contributions to the study design, drafting the manuscript and funding acquisition. All authors read and approved the final manuscript.

## Supplementary Material

Additional file 1: Figure S1 Cytokines and chemokines in bronchoalveolar lavage fluid (BAL) after segmental allergen provocation in asthmatic patients. Patients underwent BAL at baseline (B) and 24 hours after instillation with saline (S), one-tenth of the standard dose (A1/10) and standard allergen (A) in two different segments (A1 + A2). Levels of IL-5, CCL17, and CCL22 are shown. BAL from both standard allergen segments (A = A1 + A2) was pooled for CCL17 and CCL22 measurement. Bars indicate mean values (n = 11); data were analyzed by one way ANOVA. *P < .05, **P < .01 and ***P < .001. Click here for file

Additional file 2: Figure S2 Gene expression of M2 marker genes in alveolar macrophages from asthmatic patients. Asthmatic patients underwent bronchoalveolar lavage at baseline (B) and 24 h after provocation with saline (S), standard allergen (A) and one-tenth of the standard allergen dose (A1/10). RNA expression is shown as fold induction compared to the mean baseline expression in healthy patients (n = 7). Click here for file

Additional file 3: Figure S3 Gene expression of M2 marker genes in alveolar macrophages from healthy patients. Healthy subjects underwent bronchoalveolar lavage at baseline (B) and 24 h after provocation with saline (S), standard allergen (A) and one-tenth of the standard allergen dose (A1/10). RNA expression is shown as fold induction compared to the individual baseline expression in healthy patients (n = 4). Click here for file
