# Supplementary figures and images for: Impact of endobronchial allergen provocation on macrophage phenotype in asthmatics
Source: BMC Immunol. 2014 Mar 10;15:12. doi: 10.1186/1471-2172-15-12 (PMC4007705; doi:10.1186/1471-2172-15-12)

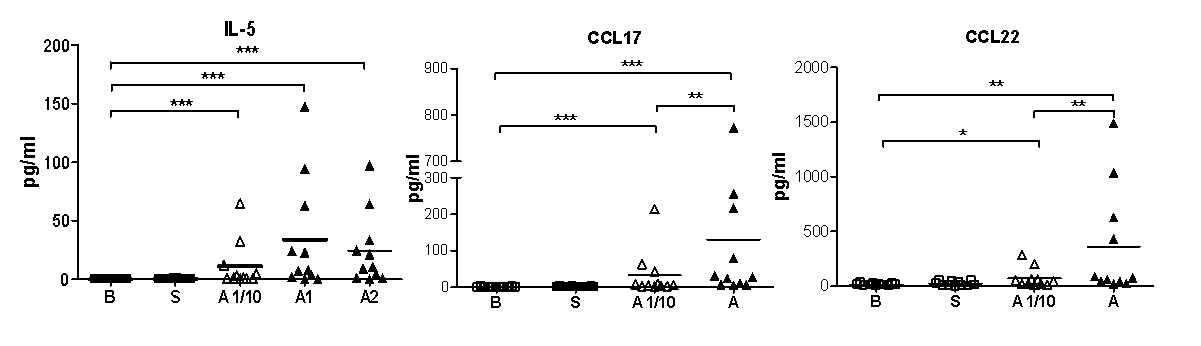

Supplement: Additional file 1: Figure S1 — Cytokines and chemokines in bronchoalveolar lavage fluid (BAL) after segmental allergen provocation in asthmatic patients. Patients underwent BAL at baseline (B) and 24 hours after instillation with saline (S), one-tenth of the standard dose (A1/10) and standard allergen (A) in two different segments (A1 + A2). Levels of IL-5, CCL17, and CCL22 are shown. BAL from both standard allergen segments (A = A1 + A2) was pooled for CCL17 and CCL22 measurement. Bars indicate mean values (n = 11); data were analyzed by one way ANOVA. *P < .05, **P < .01 and ***P < .001. [file 1471-2172-15-12-S1.tiff]

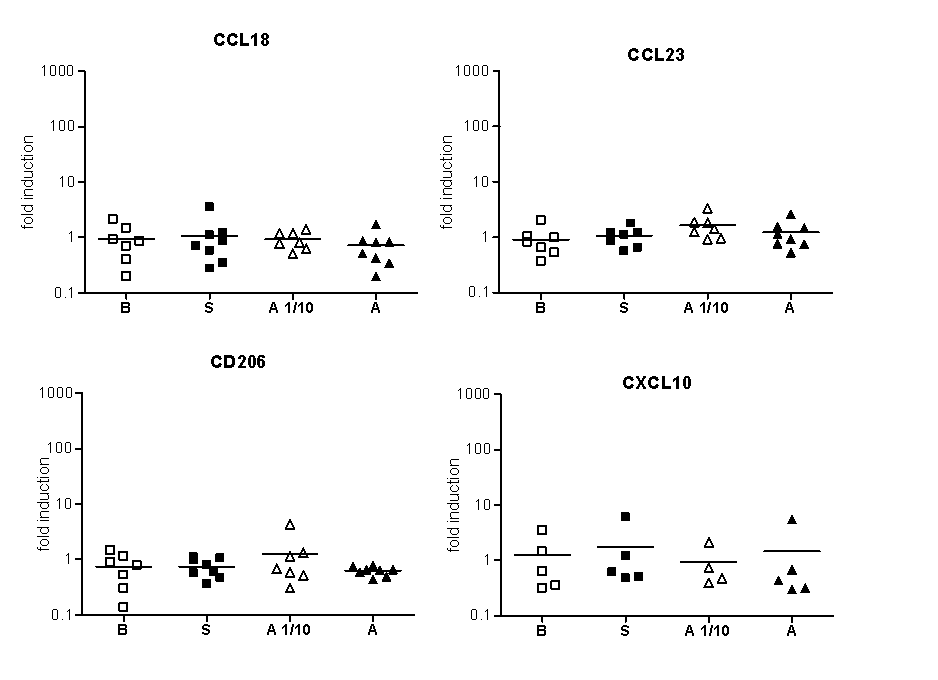

Supplement: Additional file 2: Figure S2 — Gene expression of M2 marker genes in alveolar macrophages from asthmatic patients. Asthmatic patients underwent bronchoalveolar lavage at baseline (B) and 24 h after provocation with saline (S), standard allergen (A) and one-tenth of the standard allergen dose (A1/10). RNA expression is shown as fold induction compared to the mean baseline expression in healthy patients (n = 7). [file 1471-2172-15-12-S2.tiff]

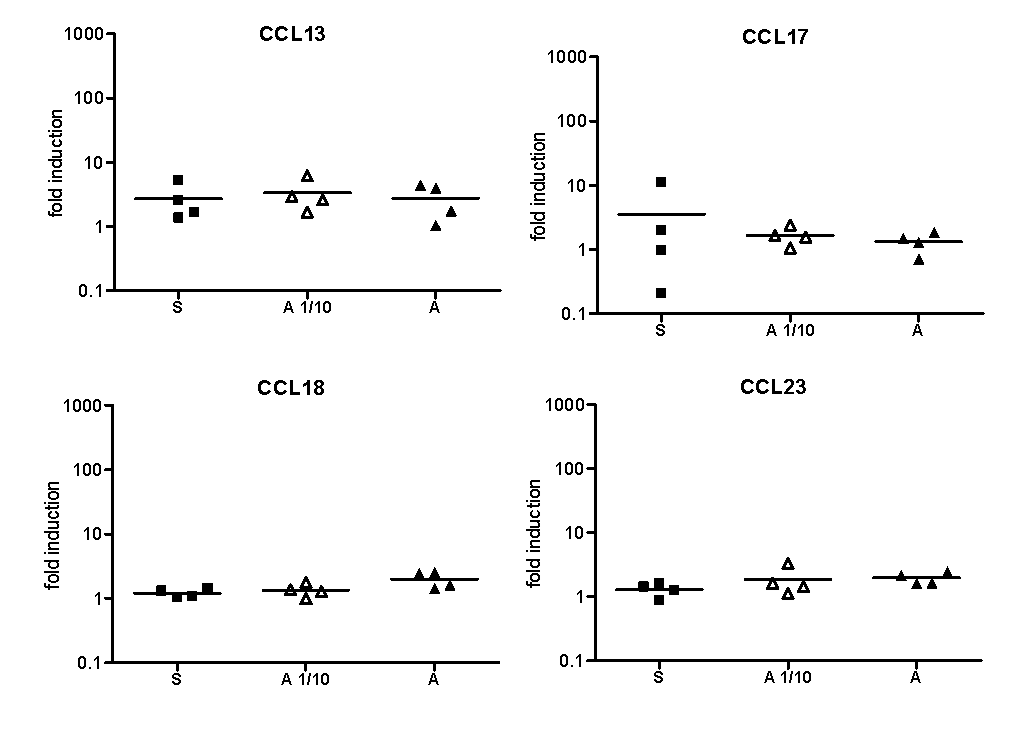

Supplement: Additional file 3: Figure S3 — Gene expression of M2 marker genes in alveolar macrophages from healthy patients. Healthy subjects underwent bronchoalveolar lavage at baseline (B) and 24 h after provocation with saline (S), standard allergen (A) and one-tenth of the standard allergen dose (A1/10). RNA expression is shown as fold induction compared to the individual baseline expression in healthy patients (n = 4). [file 1471-2172-15-12-S3.tiff]
